# Functional disability among older adults in India; a gender perspective

**DOI:** 10.1371/journal.pone.0273659

**Published:** 2022-09-14

**Authors:** Manzoor Ahmad Malik

**Affiliations:** Research Fellow Department of Humanities and Social Sciences, Indian Institute of Technology (IIT), Roorkee, Haridwar, India; Shahid Beheshti University of Medical Sciences, ISLAMIC REPUBLIC OF IRAN

## Abstract

**Introduction:**

Older adults are always at a greater risk of physical and functional health challenges. These complications result into morbidity, disability and death making them more vulnerable at later ages. Therefore, this paper will examine the functional health status among older adults and its gender perspective, along with associated risk factors.

**Materials and methods:**

Using the first round of Longitudinal ageing survey of India (2017–18). Functional disability was computed based on general and instrumental activities of daily living (ADL and IADL) (n = 20910). Functional disability was coined with individual having at least one of the limitations of these activities. Applying bivariate and multivariate analysis the present paper studied the association, gender perspective and risk factors of functional disability among older adults aged 50 and above in India.

**Results:**

Our results clearly showed the gender bias in functional disability, with greater proportion of women (52%) at risk for functional disability then men (35%). Factors like multimorbidity, depression and life satisfaction are key risk factors identified by this study that increase the likelihood of disability.

**Conclusion:**

Functional disability is key to healthy ageing and needs immediate attention given its greater concentration among the elderly, particularly women. The results reflect the substantial burden of functional disability than self-care among older adults in India and therefore indicates some significant policy interventions to reduce the likely impact of functional disability.

## Introduction

Older adults are at greater risk for multiple physical and mental health challenges, resulting into disability and death. Some of the most common problems at older ages include physical impairments, functional limitation, depressive symptoms, and poor cognition. These factors together result in the disability risking the health of the elderly in later life. Disability among older adults is attributed to many associated risks. However, four common risk factors include multimorbidity’s, depression, daily managing activities and functional limitations [1]. Research shows that older people face physiological limitations due to the ageing process, particularly functional health limitations [2]. Therefore with increase in age, these limitations result in acute morbidity conditions and functional losses (including physical and mental losses), leading to functional disability [3]. Functional disability is therefore common among older adults risking their well-being due to subsequent health loss and working abilities.

Functional limitations are a set of activities of daily living that helps an individual to work out his/her daily functions. They are broadly classified into two groups; Activities of daily living (ADL) (health, work, self-knowledge and leisure) and Instrumental Activities of daily living (IADL) (symbolizes by work and social cohesion) [4]. Therefore functional disability refers to an individual’s limitation to perform some or a set of these activities [5]. These activities at older ages are important, since they reflect the health status and provide an understanding of health care and social services through policy measures for the elderly [6].

Gender is the set of roles governed by social relations that bring reproductive distinctions between bodies into social process [7]. Gender is hardly described in disability terms, given its little bearing [8]. Gender roles are crucial to well-being since more and more women require active social participation at higher ages. However, given the functional limitation, it may likely risk their well-being and challenge their overall health and welfare [9, 10]. Women are more vulnerable to the risk of poor functional health than males and the risk is manifold particularly at upper ages [11, 12].

Women are more susceptible to poor physical health given their limitations to social and physical activities. Much of the research lacks the disability aspect from a gender perspective since gender is hardly described in disability terms [8]. Moreover, the noticeable lack of disability research from a gender aspect is also evident in the developing world, particularly among older adults from a gender perspective [13, 14].

Disability is strongly associated with age, particularly in later life. Around 700 million people are suffering from any form of disability globally, with more than 26 million from India [15]. These projected figures for India are based on the 2011 census; therefore, the numbers currently are likely to be underestimated [15]. Forty-five percent of women in India constitute the total disabled population accounting a significant proportion. Furthermore, given the rapid pace of the ageing population in India, it is likely that the disability population will significantly increase in the coming decades particularly women given their vulnerability [16, 17]. More than 40 percent elderly population in India are suffering from these functional limitations with greater proportion of them being women [18]. But very less has been examined in of how these limitations result into disability particularly at the older ages [19]. Some studies in India reported it between 30 to 40 percent, however these studies are largely under sampled. Therefore, the present paper will examine the functional disability among elderly, their gender differentials, and associated risk factors. Although studies few studies have been carried out in this context to measure the functional disability among older adults in India recently using the large-scale household surveys [16, 17]. But given the greater vulnerability of women to these disability outcomes at upper ages, this study made a detailed account of gender perspective to analyze and identify the challenges of disability among older adults in India. Moreover, given the increase in functional disability due to life style changes, ageing population and urbanization immediate attention is required for some early intervention to reduce the overall burden of disability due to functional disability in India.

## Materials and methods

### Data

This study used the first round of Longitudinal ageing survey of India (LASI) conducted in the year 2017–18. The data was collected for 72250 individuals, but this study only used the older adults aged 50 and above in this study. Therefore, the selected sample for this study was 52380 of which the older adults having any functional disability were 20910. The description of the survey, including objectives, sampling design and other detailed information, is described elsewhere in detail [18]. LASI was approved by the Ethical Review Committee of IIPS, and other partnering institutions and all participants signed informed consent at the time of participation.

### Outcome variable

This study used two variables to measure functional disability in later life, i.e., Activities of daily life (ADL) and Instrumental Activities of daily life IADL. While ADL was measured through (dressing, walking across a room, bathing, eating, getting in and out of bed, and using a toilet). IADL was measured using activity questions such as (preparing a hot meal, grocery shopping, using a telephone, managing medications, and managing money). The total number of questions included for generation the ADL score were six, whereas seven questions were used to compute the IADL score. Binary responses were recorded for measuring these variables. To generate the combined variable, we first summed the scores of both IADL and ADL variables. Functional disability was then computed as having any disability in one or more ADL/IADL activities as computed in the earlier studies [1, 6]. Finally, the dichotomous variable was calculated as (0; Not having any disability i.e., no difficulty in performing any of these activities) and (1; having any functional disability i.e., those who have recorded any disability of all the IADL and ADL activities). The detailed information about the ADL and IADL scales is given in the S1 File.

### Independent variables

The set of independent variables included in the study where age was categorized into three groups. Sex marital status, education, religion, morbidity, wealth index, place of residence and regions. Similarly, the health and risk behavior were computed like multimorbidity, self-related health, life satisfaction and depressive symptoms.

### Statistical analysis

This study carried the detailed analysis using both bivariate and multivariate methods. Gender difference was first tested using the chi-square test. Similarly, prevalence ratios were computed to examine the association between functional disability and a set of independent factors through unadjusted odds ratios. For finally studying the risk factors, logistic regression was used to study the predictors of functional disability and its risk associated with various outcomes in the study.

## Results

**Fig 1:**
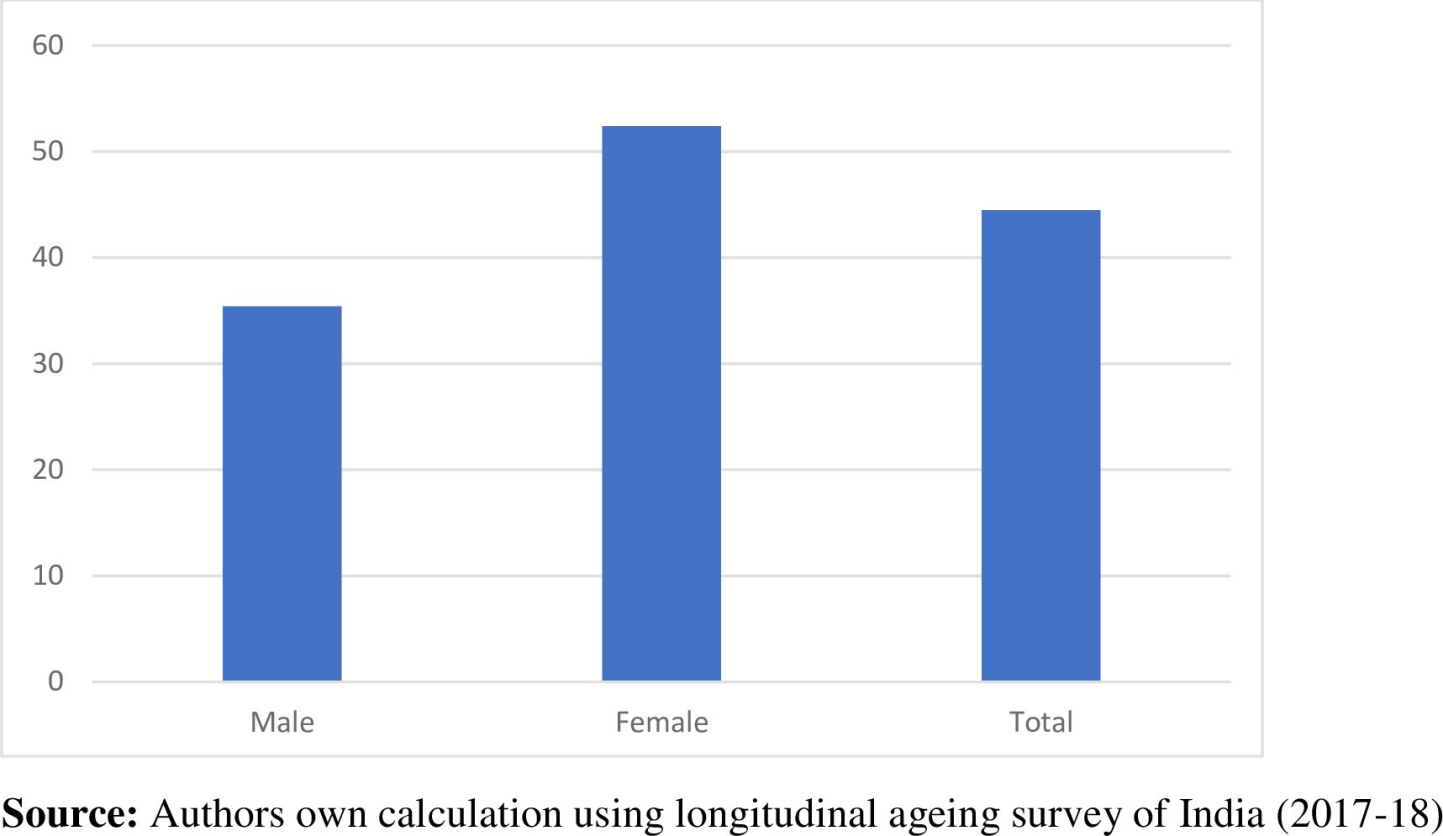
Functional disability by gender among older adults in India (LASI 2017–18).

**Fig 1** shows the prevalence of functional disability by gender among older adults aged 50 and above in India. More Than half of women (53%) at higher ages are having a functional disability as compared to 35% of males. The overall ageing population with functional Disability in India is around 44 percent.

**Fig 2:**
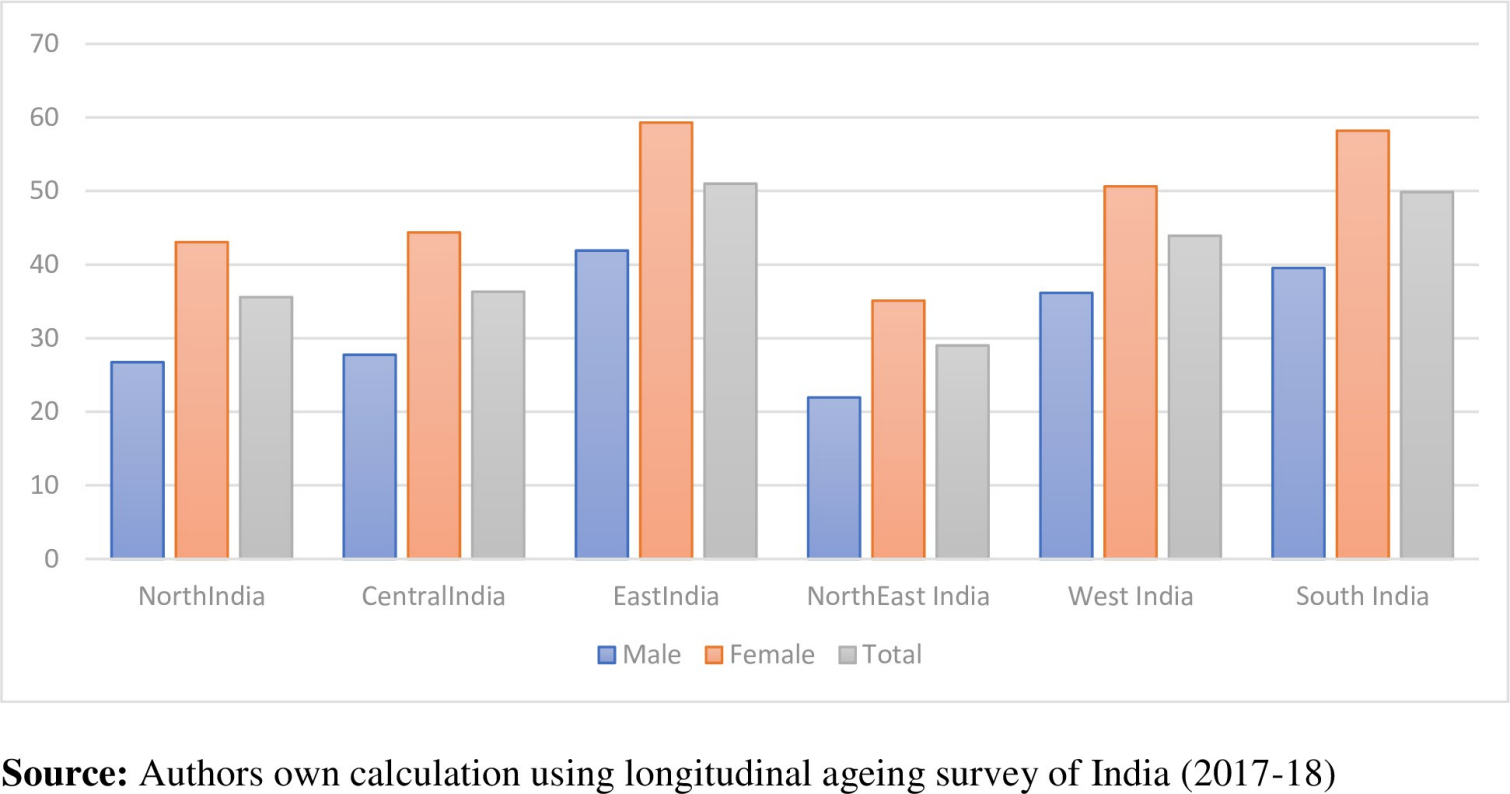
Gender differences in functional disability by regions among older adults in India (LASI 2017–18).

**Fig 2** shows the prevalence by region in India among the elderly. While females are dominating with a greater prevalence of functional disability. Highest prevalence (51%) is found in Eastern regions of India, whereas the lowest prevalence (35%) is found in the northern part of India.

**Table 1** shows the prevalence of functional disability by socio-economic and background characteristics among males and females in India. The trend of higher prevalence is clearly reflected through background characteristics, with a greater prevalence of functional disability among females. Gender differences are, therefore, highly prevalent in functional disability, as shown in Table 1. Similarly, functional disability is highly prevalent among individuals from poor socioeconomic backgrounds like income, work status, social group, religion and education. Only 23 percent of older adults have a functional disability with secondary education compared to 53 percent illiterates. Similarly, the prevalence was reported highest among adults with more than morbidity (56%), low-level satisfaction (47%) and depressive symptoms (55%).

**Table 1:** Functional disability by socio-economic and wellbeing characteristics among older adults in India (LASI -2017-18).

	*Male*	*Female*	*Total*	*Sample (N)*
**Age Group**	7626	13284	20910	50890
50–59	22.47	40.5	32.3	20910
60–69	35.38	52.44	44.45	18974
70+	53.99	70.86	62.82	12490
**Place of Residence**				
Rural	38.91	55.82	47.85	34093
Urban	27.28	44.92	36.94	18287
**Education**				
Illiterate®	46.21	57.21	53.75	25978
Lessthan5	43.84	53.73	48.03	6136
5_9yrs	30.12	41.96	34.76	11242
10+	20.75	30.5	23.55	9024
**Marital Status**				
Currently Married	33.48	45.61	38.73	37230
Widowed	47.47	61.93	58.99	13516
Others	44.03	49.26	46.71	1633
**Social Groups**				
Schedule Caste	39.37	54.95	47.77	8689
Schedule Tribe	34.65	47.03	41.5	8936
Other Backward Class	35.57	53.6	45.11	19742
General	32.68	50.32	42.13	13222
**Religion**				
Hindu	34.64	51.92	43.87	38487
Muslim	39.41	57.51	48.94	6185
Christian	33.47	46.64	41.24	5205
Other	42.76	52.58	47.94	2503
**Income Groups**				
Poorest	38.09	52.81	46.19	10492
Poorer	36.77	54.8	46.52	10667
Middle	34.78	50.2	43.08	10450
Richer	35.53	54.01	45.45	10469
Richest	31.62	49.46	40.57	10302
**Work Status**				
Currently Working	28.04	49.59	35.36	23592
Currently Not Working	49.89	61.6	55.45	14340
Never Working	44.08	49.22	48.92	14448
**Morbidity**				
No	28.89	45.87	37.56	26111
Single Morbidity	38.55	54.91	47.64	14939
More than one	48.61	63.05	56.75	11330
**Self-Related Health**				
Good	29.51	47.05	38.73	41748
Poor	57.06	68.82	63.76	9126
**Life Satisfaction**				
High	30.71	49.69	40.55	23053
Medium	38.08	51.64	45.25	12407
Low	38.53	54.28	47.47	15408
**Depressive Symptoms**				
No	30.69	47.38	39.35	37568
Yes	46.57	61.22	55.07	13322

**Source:** Authors own calculation using longitudinal ageing survey of India (2017–18)

**Table 2** shows the crude odds ratio of the socio-demographic and health outcomes associated with functional disability by males and females among the older adults in India. Crude odds ratio varied by gender but showed analogous results for both the sexes. The results from Table 2 indicated that older adults both male and female are at significantly higher odds for functional disability, but the risk among females is higher than male older adults.

**Table 2:** Association between socio-demographic and health factors with functional disability using crude odds ratio by gender among older adults aged 50 and above in India.

	COR (95%CI)	*P*.*value*	COR (95%CI)	*P*.*value*
***Age Group***				
*50–59®*				
*60–69*	1.92 (1.80–2.06)	0.000	1.63(1.55–1.73)	0.000
*70+*	4.24(3.65–4.56)	0.000	3.57(3.34–3.81)	0.000
***Place of Residence***				
*Rural ®*				
*Urban*	0.64(0.60–0.68)	0.000	0.73(0.70–0.77)	0.000
***Education***				
*Illiterate®*				
*Lessthan5*	0.77(0.71–0.84)	0.000	0.69(0.64–0.75)	0.000
*5_9yrs*	0.52(0.48–0.56)	0.000	0.50(0.47–0.54)	0.000
*10+*	0.35(0.33–0.38)	0.000	0.33(0.31–0.37)	0.000
***Marital Status***				
*Currently Married®*				
*Widowed*	1.97(1.81–2.13)	0.000	1.90(1.81–1.99)	0.000
*Others*	1.41(1.21–1.64)	0.000	1.06(0.92–1.22)	0.480
***Social Groups***				
*Schedule Caste®*				
*Schedule Tribe*	0.70(0.64–0.77)	0.000	0.66(0.61–0.72)	0.000
*Other Backward Class*	0.91(0.84–0.98)	0.012	0.97(0.90–1.04)	0.302
*General*	0.80(0.74–0.87)	0.000	0.88(0.82–0.95)	0.002
***Religion***				
*Hindu®*				
*Muslim*	1.02(0.93–1.11)	0.736	1.21(1.13–1.31)	0.000
*Christian*	0.64(0.57–0.70)	0.000	0.59(0.55–0.64)	0.000
*Other*	1.08(0.95–1.22)	0.251	0.87(0.77–0.97)	0.000
***Income Groups***				
*Poorest®*				
*Poorer*	0.94(0.86–1.02)	0.140	0.98(0.91–1.05)	0.582
*Middle*	0.86(0.79–0.94)	0.000	0.95(0.89–1.03)	0.220
*Richer*	0.85(0.78–0.92)	0.000	0.95(0.88–1.02)	0.143
*Richest*	0.79(0.72–0.86)	0.000	0.86(0.80–0.93)	0.000
***Work Status***				
*Currently Working®*				
*Currently Not Working*	2.54(2.40–2.70)	0.000	1.78(1.66–1.90)	0.000
*Never Working*	1.61(1.41–1.84)	0.000	1.12(1.06–1.18)	0.000
***Morbidity***				
*No®*				
*Single Morbidity*	1.54(1.45–1.65)	0.000	1.40(1.32–1.47)	0.000
*Multi Morbidity*	2.11(2.10–2.43)	0.000	1.92(1.81–2.04)	0.000
***Self-Related Health***				
*Good®*				
*Poor*	3.32(3.10–3.55)	0.000	2.63(2.47–2.80)	0.000
***Life Satisfaction***				
*High®*				
*Medium*	1.25(1.17–1.34)	0.000	1.19(1.12–1.26)	0.000
*Low*	1.43(1.34–1.52)	0.000	1.29(1.22–1.37)	0.000
***Depression***				
*No®*				
*Yes*	1.99(1.87–2.12)	0.000	1.90(1.80–2.01)	0.000

**Source:** Authors own calculation using longitudinal ageing survey of India (2017–18)

**Note:** COR; Crude Odds Ratio. Not having any functional disability is the reference category; 95% confidence interval in parentheses; significance level: ®; Represents reference category for predictors.

**Table 3** shows the results of multiple logistic regression results. Three logistic models were run for males, females and overall samples respectively to study the odds of various risk factors. Overall, the risk is significant and higher among females. However, some factors were insignificant as well, such as wealth status, religion, and social groups.

**Table 3:** Predictors of functional disability using adjusted odds ratio by gender and overall among older adults aged 50 and above in India.

	*Model 1*		*Model 2*		*Model 3*	
	AOR (95%CI)	P.value	AOR (95%CI)	P.value	AOR (95%CI)	P.value
**Age Group**						
50–59®						
60–69	1.50(1.39–1.61)	0.000	1.37 (1.29–1.45)	0.000	1.34(1.28–1.40)	0.000
70+	2.53(2.32–2.76)	0.000	2.37(2.20–2.56)	0.000	2.12(2.01–2.24)	0.000
**Place of Residence**						
Rural ®						
Urban	0.69(0.64–0.74)	0.000	0.81(0.77–0.86)	0.000	0.78(0.74–0.81)	0.000
**Education**						
Illiterate®						
Lessthan5	0.79(0.72–0.87)	0.000	0.71(0.65–0.78)	0.000	0.68(0.64–0.72)	0.000
5_9yrs	0.57(0.53–0.62)	0.000	0.58(0.53–0.62)	0.000	0.50(0.47–0.52)	0.000
10+	0.39(0.36–0.43)	0.000	0.41(0.37–0.45)	0.000	0.33(0.31–0.35)	0.000
**Marital Status**						
Currently Married®						
Widowed	1.18(1.07–1.29)	0.001	1.21(1.14–1.28)	0.000	1.38(1.31–1.44)	0.000
Others	1.31(1.10–1.56)	0.003	1.07(0.92–1.25)	0.385	1.23(1.09–1.38)	0.000
**Social Groups**						
Schedule Caste®						
Schedule Tribe	0.96(0.85–1.07)	0.452	0.86(0.78–0.94)	0.002	0.92(0.86–0.99)	0.033
Other Backward Class	1.02(0.93–1.11)	0.706	1.04(0.96–1.12)	0.320	1.05(0.99–1.11)	0.117
General	1.04(0.95–1.15)	0.398	1.10(1.01–1.19)	0.032	1.09(1.02–1.16)	0.008
**Religion**						
Hindu®						
Muslim	0.91(0.82–1.01)	0.050	1.15(1.06–1.25)	0.001	0.98(0.92–1.04)	0.520
Christian	0.63(0.55–0.71)	0.000	0.69(0.63–0.77)	0.000	0.68(0.62–0.73)	0.000
Other	1.07(0.92–1.23)	0.389	0.93(0.82–1.05)	0.224	0.95(0.87–1.05)	0.326
**Income Groups**						
Poorest®						
Poorer	0.97(0.89–1.07)	0.568	1.03(0.95–1.12)	0.449	1.01(0.95–1.08)	0.676
Middle	0.92(0.84–1.01)	0.088	1.01(0.92–1.09)	0.996	0.98(0.92–1.04)	0.456
Richer	0.91(0.82–1.01)	0.046	1.03(0.95–1.12)	0.503	0.99(0.93–1.06)	0.828
Richest	0.9(0.81–0.99)	0.038	1.02(0.94–1.12)	0.594	1.01(0.93–1.06)	0.823
**Work Status**						
Currently Working®						
Currently Not Working	1.57(1.47–1.69)	0.000	1.17(1.09–1.26)	0.000	1.48(1.41–1.56)	0.000
Never Working	1.04(0.90–1.21)	0.594	0.91(0.86–0.98)	0.008	1.34(1.27–1.40)	0.000
**Morbidity**						
No®						
Single Morbidity	1.42(1.32–1.53)	0.000	1.32(1.24–1.40)	0.000	1.38(1.32–1.45)	0.000
Multi Morbidity	1.94(1.79–2.11)	0.000	1.76(1.64–1.89)	0.000	1.88(1.78–19.8)	0.000
**Self-Related Health**						
Good®						
Poor	2.16(2.01–2.34)	0.000	1.90(1.78–2.03)	0.000	2.01(1.91–2.12)	0.000
**Life Satisfaction**						
High®						
Medium	1.13(1.05–1.22)	0.000	1.08(1.01–1.15)	0.022	1.09(1.04–1.15)	0.000
Low	1.07(0.99–1.15)	0.077	0.99(0.93–1.05)	0.777	1.01(0.97–1.06)	0.605
**Depression**						
No®						
Yes	1.57(1.46–1.68)	0.000	1.54(1.46–1.64)	0.000	1.54(1.48–1.61)	0.000

**Source:** Authors own calculation using longitudinal ageing survey of India (2017–18)

**Note:** AOR; Adjusted Odds Ratio. Not having any functional disability is the reference category; 95% confidence interval in parentheses; ®; Represents reference category for predictors.

**Model 1** shows the odds of various socio-economic and demographic factors among males aged 50 and above. Male respondents who have poor self-related health have a risk of functional disability more than those who are not having poor self-related health (OR = 2.16, 95% CI = 2.00–2.34). Similarly, factors like age, low level of satisfaction and work status were associated with functional disability.

**Model 2** studies the risk factors associated with functional disability among females of age 50 and above. Factors like morbidity, self-related health and depression were significant and positively associated. Although income was positively associated, the results were insignificant. Similarly, education was significant but less likely to be associated with the risk of functional disability.

**Model 3** examines the impact of risk factors on all samples, and the results reflect the significant association. Having any morbidity meant a higher rate of functional disability (OR = 1.1.38, 5% CI = 1.32–1.45). Respondents with any depressive symptoms have higher odds of functional disability (OR = 1.54, 95% CI = 1.48–1.61). Similarly, other risk factors were also strongly associated with the risk of functional disability.

## Discussion

Older adults are at greater risk of any disability-related to physical, functional or mental health [20]. Increasing age limits the physical and functional health outcomes at higher ages and thus risks the overall wellbeing of older adults [21, 22]. This study attempted to measure the gender dimension of functional disability among older adults aged 50 and above in India. Functional disability was significantly higher among the women’s compared to older men in India. Our results first tried to understand the difference in functional disability across the gender and found the significant differences. Women are at greater risk of functional disability, given the various risk factors related to them (socio-economic and wellbeing) and analogous with earlier studies [23].

Older adults suffering from any health complication such as multimorbidity or having any depressive symptoms, including socio-demographic factors like age, place of residence, education, and income, are strongly associated with greater risk of functional disability. These results were coherent to earlier studies carried out in this context [24]. Poor socio-economic and wellbeing settings risks the over all wellbeing of the older adults and makes them more vulnerable to perform certain functions due lack of proper nutritional intake, heavy physical and psychological workload, and availability of basic amenities to enhance their wellbeing [25, 26]. Older adults with poor self-related health and low life satisfaction are also at greater risk of having any functional disability. The differences are notably higher among females given the greater likelihood of these disability risks, given their vulnerability to socio-economic and wellbeing settings [27]. Various studies in this context have been carried out in India and worldwide to examine functional disability and its gender differentials [28, 29]. A study carried out among seniors in Bangladesh showed that female suffer more due to functional limitation than their male counterparts at upper ages [13]. Similarly a study carried out in Brazil also showed women’s at greater risk [30]. There are similar other studies that have showed the gender differentials in functional disability however, some studies have also shown that functional disability was almost same among males and females in different countries [3]. Studies carried out in India were so far mainly based on primary surveys conducted in various subpopulations [31]. But the recent studies using secondary data have found significant risk associated with functional disability among older adults particularly females [17, 32].

Factors such as wealth and residence are significantly associated with the risk of functional disability. The findings corroborate with earlier studies which have examined the differences and risk factors in functional disability [28, 33]. Moreover older adults are at greater risk of being affected by impaired functions that involve a lack of ADL and IADL, resulting in greater risk for disability and death [34]. Our results confirmed that prevalence is likely affected by socio-demographic factors, which increase the risk for loss of functional health among the older adults, particularly women as identified by few various studies in different subpopulations [23, 35, 36].

Morbidity is one of the critical risk factors that result in greater levels of disability [37]. The findings from the paper confirmed that older adults with multimorbidity have higher levels of functional disability, similar to the findings of other studies [37, 38]. Therefore, given the significant association of morbidity with functional disability, there is a greater need for reducing the likelihood of chronic diseases through access to healthcare services and financing among the older adults in India [39].

Depressive symptoms were also associated with greater levels of functional disability in both men and women. However, the risk was higher among males, which was a significant finding of this study. Males are likely at greater risk for depressive symptoms, therefore it increases the risk for functional disability among them [40]. Moreover, the low life of satisfaction also enhances the functional disability due to challenges social cohesion given their poor socio-economic and living conditions. The results were coherent with earlier studies [41, 42].

This study underscores the urgency for policy interventions that can lower the burden for functional disability from a gender perspective. Moreover, there is a need for increasing incentives for older women to lower the disability burden and make them socially more inclusive at older ages. Social programs can be also critical in this aspect to aim for greater focus on enhancing the older adults needs and improving the daily activities of older adults and their well-being.

To sum up, this study shows the apparent differences in functional disability with women being more vulnerable given the challenges they face at older ages. Therefore, women-centric policies are vital to mitigating this health crisis and making women more inclusive of studying the health care services and well-being among them at older ages. There is an urgent need for health care services provision and social security incentives in India that can address geriatric care and provide community care with a particular focus on older women. Policy incentives are required to avert the secondary disability crisis in India, which otherwise will have a significant impact on successful and healthy ageing in the country.

Functional disability is key to healthy ageing and needs immediate attention given its greater concentration among the elderly, particularly women. The results reflect the more significant burden of functional disability than self-care among older adults in India and therefore indicates some significant policy interventions to reduce the likely burden of functional disability among the older adults in India.

### Limitations

Despite providing the conclusive evidence, this study has some potential limitations which might have affected the overall results of the study. Information related variables such as health, morbidity and depression were mainly self-reported so there maybe the likelihood of recall bias in this study. This study also computed functional disability as the binary variable, which can disregard the varying degrees of incapacity of individuals and repercussions in various areas of lives, as well as real limitations and adaptability. Lastly this study could not include the various other risk factors of disability which might have provided the better study results in this context.

## Supporting information

S1 File (DOCX)
