# Supplementary material for: Functional disability among older adults in India; a gender perspective
Source: PLoS One. 2022 Sep 14;17(9):e0273659. doi: 10.1371/journal.pone.0273659 (PMC9473438; doi:10.1371/journal.pone.0273659)
Supplement: S1 File — (DOCX) [file pone.0273659.s001.docx]

**Supplementary File**

**Variables in the Study**

**Data**

This study used the data from the first round of Longitudinal ageing survey of India conducted in the year 2017-18. LASI is a survey on older adults covering the broad range of issues varying from socio-economic to physical and mental health of older adults aged 45 and above in India. We included the sample of 50 and above only which is around 52380 with 20910 older adults having any functional disability.

**Variables in the Study**

**Work Status;** Work status denotes the current work status of the older adults which was computed based on the question “Are you currently working”. It includes also those working at the time of the survey or those who are temporarily laid off, sick, or in training. The variable was then made trichotomous with (0= Currently Not Working), (1= Currently Working) and (2= who has never worked). Never worked was based on the computed who does not provided any response to the above question.

**Self-Related Health (SRH);** Self-rated health was having a scale of 1–5 “poor to excellent” and was categorized as 0 “good” (representing good, very good and excellent) and 1 “poor” (representing poor or fair) in our paper

**Multi-Morbidity;** Multi-morbidity conditions refer to the presence of two or more chronic diseases which include hypertension, chronic heart diseases, stroke, any chronic lung disease, diabetes, cancer or malignant tumor, any bone/joint disease, any neurological/psychiatric disease or high cholesterol. The variable in the paper was computed on the following questions based on the binary score of yes and no from these morbidity related questions. The variable was then categorized into three groups with 0= No morbidity, 1= Single morbidity and 2 = more than one

**Depressive Symptoms; Depressive symptoms were measured based on the CES-D scale which included 10 items with** seven negative symptoms (trouble concentrating, feeling depressed, low energy, fear of something, feeling alone, bothered by things, and everything is an effort), and three positive symptoms (feeling happy, hopeful, and satisfied). Response options included rarely or never (< 1 day), sometimes (1 or 2 days), often (3 or 4 days), and most or all of the time (5-7 days) in a week prior to the interview. For negative symptoms, rarely or never (< 1 day), and sometimes (1 or 2 days) were scored zero, and often (3 or 4 days) and most or all of the time (5-7 days) categories were scored one. Scoring was reversed for positive symptoms. The overall score ranges from zero to 10 and score of four or more are used to calculate the prevalence of depressive.

**Cognition Score;** Cognition score was measured by the number of words recall. To measure this a scale of 0-10 was prepared representing higher score for better cognitive ability based on words recalled 2 and above. Whereas poor was computed based on less than 2 words recalled.

**Activities of Daily Living (ADL);** ADL was computed based on a set of six questions which includes dressing, putting on chapels or shoes, walking across a room, bathing, eating difficulties, getting in or out of bed (any one or more). A combined score was generated based on the set of questions which was later categorized into none, single and more than one activities respectively

**Instrumental Activities of Daily Living (IADL);** This was based on a set of 7 questions with binary response of yes and no. These questions include preparing a hot meal, shopping for groceries, making telephone calls, taking medications, doing work around the house or garden, managing money and getting around or finding address in unfamiliar place. Again a score was generated based on these questions and then categorized into three groups with, No, Single and more than one respectively.

**Functional Disability**: Functional disability was measured combining the ADL and IADL. Individuals having anyone of the limitations was termed as disable. The variable was then computed as binary variable with 0 representing no disability and 1 representing having any functional disability.

**Life satisfication** Life satisfication was computed based on a set of questions in which respondents were asked to agree or disagree with life events. Respondents were asked to say “how much you strongly agree, somewhat agree, slightly agree, neither agree nor disagree, slightly disagree, somewhat disagree or strongly disagree with the following set of questions that include ideal life, life conditions, life satisfication, achievements and about the change that an elderly can bring”. Based on the above set of responses a score was generated 5-35 which was then categorized into three categories (5/20 = 0 “Low”) (21/25=1 “Medium”) and (26/35=2 “High”). Finally the low satisfication was used in the index computation in this study.

**Wealth Index**: Measures of household wealth, as indexed by housing conditions and assets. A detailed questionnaire was asked to respondents regarding their housing and assets and then the combined score of wealth index was generated to examine the economic dimension in the survey.

**Other Covariates**

All the other independent variables such as religion, social group, marital status and place of residence were used as per the information taken about them in the survey.
